# Antroquinonol Targets FAK-Signaling Pathway Suppressed Cell Migration, Invasion, and Tumor Growth of C6 Glioma

**DOI:** 10.1371/journal.pone.0141285

**Published:** 2015-10-30

**Authors:** Varadharajan Thiyagarajan, May-Jywan Tsai, Ching-Feng Weng

**Affiliations:** 1 Department of Life Science and the Institute of Biotechnology, National Dong Hwa University, Hualien, 97401, Taiwan; 2 Neural regeneration Laboratory, Neurological Institute, Taipei Veterans General Hospital, Taipei, 11217, Taiwan; INSERM, FRANCE

## Abstract

Focal adhesion kinase (FAK) is a non-receptor protein tyrosine that is overexpressed in many types of tumors and plays a pivotal role in multiple cell signaling pathways involved in cell survival, migration, and proliferation. This study attempts to determine the effect of synthesized antroquinonol on the modulation of FAK signaling pathways and explore their underlying mechanisms. Antroquinonol significantly inhibits cell viability with an MTT assay in both N18 neuroblastoma and C6 glioma cell lines, which exhibits sub G1 phase cell cycle, and further induction of apoptosis is confirmed by a TUNEL assay. Antroquinonol decreases anti-apoptotic proteins, whereas it increases p53 and pro-apoptotic proteins. Alterations of cell morphology are observed after treatment by atomic force microscopy. Molecular docking results reveal that antroquinonol has an H-bond with the Arg 86 residue of FAK. The protein levels of Src, pSrc, FAK, pFAK, Rac1, and cdc42 are decreased after antroquinonol treatment. Additionally, antroquinonol also regulates the expression of epithelial to mesenchymal transition (EMT) proteins. Furthermore, antroquinonol suppresses the C6 glioma growth in xenograft studies. Together, these results suggest that antroquinonol is a potential anti-tumorigenesis and anti-metastasis inhibitor of FAK.

## Introduction

Focal adhesion kinase (FAK), a protein tyrosine kinase, localizes to focal adhesions and is involved in several cellular functions such as survival, invasion, motility, adhesion, metastasis, proliferation, and angiogenesis [1]. FAK is autophosphorylated at tyrosine 397, which results in a high binding affinity site for the SH2 domain of the src family kinases [2, 3]. The mutually activated FAK/Src complex then activates a cascade of phosphorylation events in new protein—protein interactions to trigger several signaling pathways that eventually lead to different cellular responses. FAK can recruit SOS into the complex that activates the downstream Ras-MAPK pathway and/or transduces the signal through the activation of the PI3K-Akt cascade [4–6]. Recent work shows the active Src/FAK complex stimulates Rac1 activity through the recruitment and the phosphorylation of the scaffolding protein p130Cas [7]. Rac1 and Cdc42 GTPase regulate the assembly of multi-molecular focal complexes associated with actin stress fibers, filopodia, and lamellipodia, which are necessary for cell motility and are indirectly activated by Src tyrosine kinase [8, 9]. From the inhibition of the apoptotic response, FAK/Src activation may promote the anchorage independent transformation and growth of tumor cells [10, 11].

*Antrodia camphorata* (*A*. *camphorata*) is a new basidiomycete in the Polyporaceae (Aphyllophorales) family that has been identified as a distinct species of the genus Antrodia, Taiwan [12]. *A*. *camphorate* is used to treat the symptoms of various diseases such as abdominal pain, diarrhea, itching of skin, and hypertension [13]. Previous studies have demonstrated the growth inhibitory effects of *A*. *camphorate* on cancers such as ovarian cancer, breast cancer, bladder carcinoma, and prostate cancer [14–17]. Recently, ethanol extracts of *A*. *camphorata* mycelia (SACE and Fraction-6) can induce apoptosis of non-small cell lung cancer (NSCLC) cells by down-regulating the synthesis of RhoGDI-alpha, galectin-1, human calpain small (regulatory) subunits, and eIF5A [18, 19]. The biological activity of *A*. *camphorate* is dominantly carried out by high amounts of terpenoids, polysaccharides, and succinic and maleic acid derivatives [20]. All the tested extracts of *A*. *camphorata* possess an anti-inflammatory effect from the suppression of nitric oxide (NO) by reducing the inducible nitric oxide synthase (iNOS) expression in an activated macrophage or microglia [21]. Antroquinonol isolated from *A*. *camphorate* has been reported to be antitumorigenic on different cancer cells. Antroquinonol displays anticancer activity against hepatocellular carcinoma cells through AMPK activation [22] and the inhibition of the mTOR translational pathway in A549 lung cancer cell [23]. In the present study, we demonstrate that synthetic antroquinonol-induced apoptosis, suppressed FAK signaling in both N18 neuroblastoma and C6 glioma cell lines, and inhibited C6 glioma cancer growth in mouse xenograft tumor models.

## Materials and Methods

### Chemicals and antibodies

The primary antibodies for Bak, pFAK, Bad, Bax, Bcl2, and PARP were purchased from Cell Signaling (Danvers, MA, USA). Rac1 and Cdc42 antibodies were obtained from Millipore (Billerica, MA, USA). The FAK antibody was obtained from Santa Cruz Biotechnology (Santa Cruz, CA, USA), p53 and NF-kB antibodies were purchased from Neomarkers (Fremont, CA, USA), the vimentin and E-cadherin antibody were obtained from BD Biosciences (CA, USA), and β-catenin, Smad 2, and 3 antibodies were obtained from Genetex (Irvine, CA, USA). The antibody for β-actin was obtained from Sigma (USA), the anti-mouse and anti-rabbit IgG horseradish peroxidase-conjugated secondary antibodies were purchased from GE Healthcare (UK). PF-431396 was purchased from Sigma Aldrich. Synthetic antroquinonol [24] was obtained from Dr. Chin Po Chen (Department of Chemistry, NDHU, Hualien, Taiwan).

### Cell culture

Rat glioma C6 was derived from a rat glial tumor induced by N-nitrosomethylurea [25], were obtained from Sigma Aldrich (MO,USA). N18 is a clonal line produced from the mouse neuroblastoma C1300 (Mouse strain A/Jax) [26], were obtained from public health England (Salisbury, UK). BEAS-2B from human bronchial epithelium normal cells [27], were purchased from Sigma Aldrich (USA). The C6 cell line was cultured in DMEM supplemented with 15% horse serum, 2.5% FBS, and 1% (100 U/mL penicillin and 100 μg/mL streptomycin) antibiotics at 37°C in a humidified atmosphere of 5% CO_2_. N18 and BEAS-2B cells were cultured in DMEM supplemented with 10% FBS and 1% antibiotics. These cells were tested and authenticated by the provider.

### Cell viability assay

The 3-(4,5-dimethylthiazol-2-yl)-2,5-diphenyltetrazolium bromide (MTT, Invitrogen) assay is a colorimetric technique that is performed to analyze cell proliferation. Cells (1 x 10^4^ cells) were seeded in 96-well plates overnight. Cells were treated with various concentrations of the drug for an additional 24 h, and then 20 μL (5 mg/mL) of MTT solution was added per well and further incubated for 4 h. The medium was removed, and formazan was solubilized by adding 100 μL/well of DMSO (Sigma-Aldrich) and the OD was measured at 570 nm using a microplate reader (ELISA reader, Thermo Labsystems). The percentage of viable cells was compared to untreated control cells.

### Cell cycle analysis

The cells (2 x 10^5^) were seeded in 6-well plates and incubated overnight. Cells were treated with various concentrations of antroquinonol (5 and 10 μM) for an additional 24 h. After treatment, cells were harvested and fixed with ice cold 70% ethanol for 1 h at -20°C. Next, the cells were washed twice with cold PBS and resuspended in 1 mL (v/v) of staining solution containing 20 μg/mL propidium iodide, 0.1% Triton X-100, and 0.2 mg/mL RNase (Bionovas). They were incubated in a water bath at 37°C for 30 min. Lastly, the results were analyzed using a flow cytometer (Beckman Coulter).

### TUNEL assay

A terminal nucleotidyl transferase-mediated nick end labeling (TUNEL) assay was performed according to the manufacturer’s instructions (Promega Corporation, Madison, WI, USA). The cells were seeded in chamber slides (Nalge Nunc International, Rochester, NY, USA) and treated with 10 μM antroquinonol for 24 h. After treatment, the cells were fixed in 3.7% formaldehyde at room temperature (RT) for 25 min, and permeabilized in 0.1% Triton^®^ X-100 at RT for 5 min. The cells were incubated with the TUNEL reaction mixture (Equilibration Buffer, Nucleotide Mix, and rTdT Enzyme) for 60 min at 37°C in a humidified atmosphere, and DNA fragmentation was detected immuno-histochemically using the DeadEnd^TM^ Fluorometric TUNEL System.

### Tapping-mode atomic force microscopy (TM-AFM) scanning

This experiment was measured as previously described [28] with slight modification. Cells were seeded on a cover slip and incubated for 24 h. After treatment with different concentrations of antroquinonol for 24 h, cells were washed with PBS, and fixed with 1% glutaraldehyde for 5 min. The cells were imaged with a bio-atomic force microscope (Bio-AFM, Nanowizard, JPK, Germany) that was mounted on an inverted microscope, TE-2000-U (Nikon, Tokyo, Japan). Silicon nitride non-sharpened probes with a nominal cantilever force constant of 0.06 Nm-1 (DNP-20, Veeco, CA, USA) were used. Imaging was performed using contact mode. Line scan rates varied from 0.5 to 2 Hz.

### *In vitro* FAK [pY397] assay

The pFAK (Y397) kit was purchased from Invitrogen (www.invtrogen.com) and the procedure was performed according to the manufacturer’s protocol. The absorbance of samples was read at 450 nm and the readout was plotted on a graph against standard concentrations. One unit is equivalent to the amount of FAK (pY397) autophosphorylated at 300 pg of total FAK protein.

### Gelatin zymography for MMP2 and MMP9

The activities of MMP2 and MMP9 were determined by gelatin zymography. Cells (2 x 10^5^) were seeded in 6-well plates and incubated until reaching 80% confluence. Cells were starved in DMEM containing 0.1% BSA for 6 h, and then they were treated with various concentrations of antroquinonol for 24 h. The supernatants were collected and the protein concentration was quantified by Bradford dye (Bio-Rad). Next, 8% SDS-PAGE gels containing 10% gelatin were prepared for the detection of MMP2 and MMP9. After electrophoresis, gels were washed twice with washing buffer containing 2.5% Triton X-100, and then incubated in developing buffer (0.05 M Tris-HCl, pH 8.8, 5 mM CaCl_2_, 0.02% NaN_3_) at 37°C for 16 h. Finally, the gel was stained in 0.1% Coomassie blue R-250 (Bio-Rad) for 4 h, and then destained by fixing buffer (45% methanol, 10% acetic acid). The gels were scanned using an Epson scanner and quantified using multi-gauge software (Fujifilm).

### Western blotting

The cells (5 x 10^5^) were seeded in a 6 cm dish, grown until 80% confluent, and then incubated with various concentrations of antroquinonol for 24 h. Cells were collected and lysed with RIPA buffer. Protein samples were loaded and separated with SDS-PAGE then transferred to the PVDF membrane (PerkinElmer, Turku, Finland). The membranes were incubated with appropriate primary antibodies at 4°C overnight. The membranes were washed 3 times with TBST, and then incubated with horseradish peroxidase (HRP)-conjugated secondary antibodies at RT for 1 h. Finally, the membranes were exposed to ECL reagents (PerkinElmer) for 1 min and the results were analyzed with LAS-3000 film (Fujifilm, Tokyo, Japan). Beta actin was used as an internal control.

### Molecular modeling and docking

The experiment was performed as described previously [28]. The ligand (antroquinonol) was docked into the active site using the ‘Ligand Fit’ option. The docked proteins with low energy were recorded and validated.

### Animal xenograft tumor model

Six-week-old athymic male nude mice were purchased from LASCO (Charles River technology, Taipei, Taiwan). All animal experiments were performed in accordance with the “Guide for the Care and Use of Laboratory Animals” of National Dong-Hwa University (Hualien, Taiwan). A total of 5x10^5^ C6 cells were injected subcutaneously on the back of nude mice. When the tumor volume reached an average volume of approximately 200 mm^3^, the mice were randomly divided into two groups: 5 mice received intraperitoneal injection of normal saline as control (n = 5) and 5 received intraperitoneal administration of Antroquinonol (0.082 mg/kg/daily, n = 5) for 10 days. The mice were sacrificed and tumors were excised and weighed. Major organs such as liver, kidney, spleen, and tumor were subjected to histological analysis.

### Statistical analysis

The results are presented as the mean ± SD. Data were analyzed with a one-way ANOVA Tukey test for multiple-group comparisons. The significant differences (*p < 0.05), (**p < 0.01), and (***p < 0.001) between the means of control and the treatment groups were analyzed.

## Results

### Cell viability

**Fig 1:**
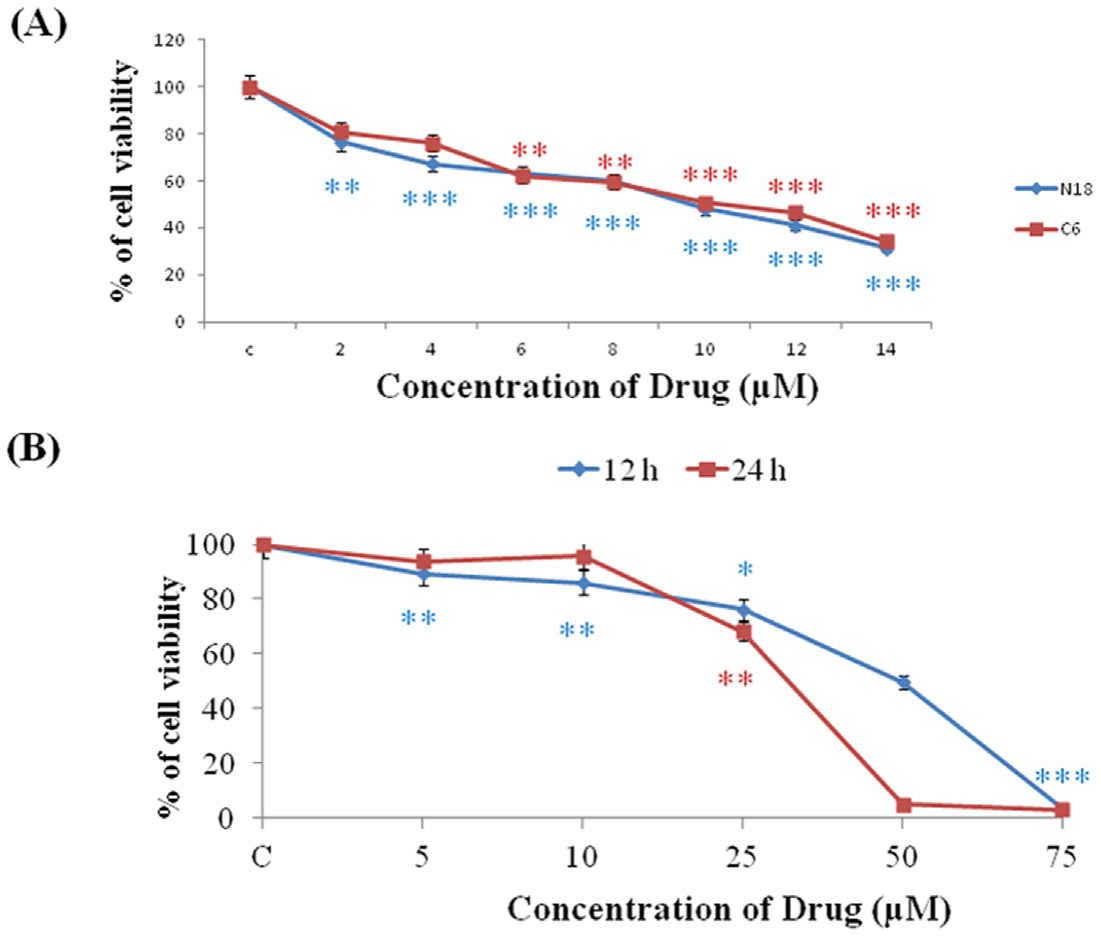
The anti-proliferative effect of antroquinonol on N18 and C6 cells. The cell viability was determined by an MTT assay. (A) The N18 and C6 cells were treated with various concentrations of the antroquinonol for 24 h, and the surviving cells were determined and presented as a percentage of the untreated cells as controls. (B) The BEAS-2B cells were treated with various concentration of drug for 12 and 24 h. The data shown are the mean from three independent experiments. **p < 0.01 and ***p < 0.001 when compared with the untreated controls.

Fig 1A illustrates the incubation of cells with the desired concentrations of antroquinonol for 24 h to determine the cytotoxic effects of antroquinonol on both the C6 and N18 cell lines, and an MTT assay was performed to analyze cell viability. After 24 h of treatment, the cell viability was significantly decreased with an increasing concentration of antroquinonol. Antroquinonol reached the IC_50_ value at a concentration of 10 μM. Hence, this concentration was applied for the subsequent experiments. The cytotoxic effect was further validated on BEAS-2B Human bronchial epithelium normal cell lines for 12 and 24 h, intriguingly antroquinonol treatment did not exhibit any toxicity (0 to 25 μM) concentration (Fig 1B). This result implies that antroquinonol was more potent cytotoxicity to cancer cells compared with the normal cells.

### Cell cycle (TUNEL assay)

**Fig 2:**
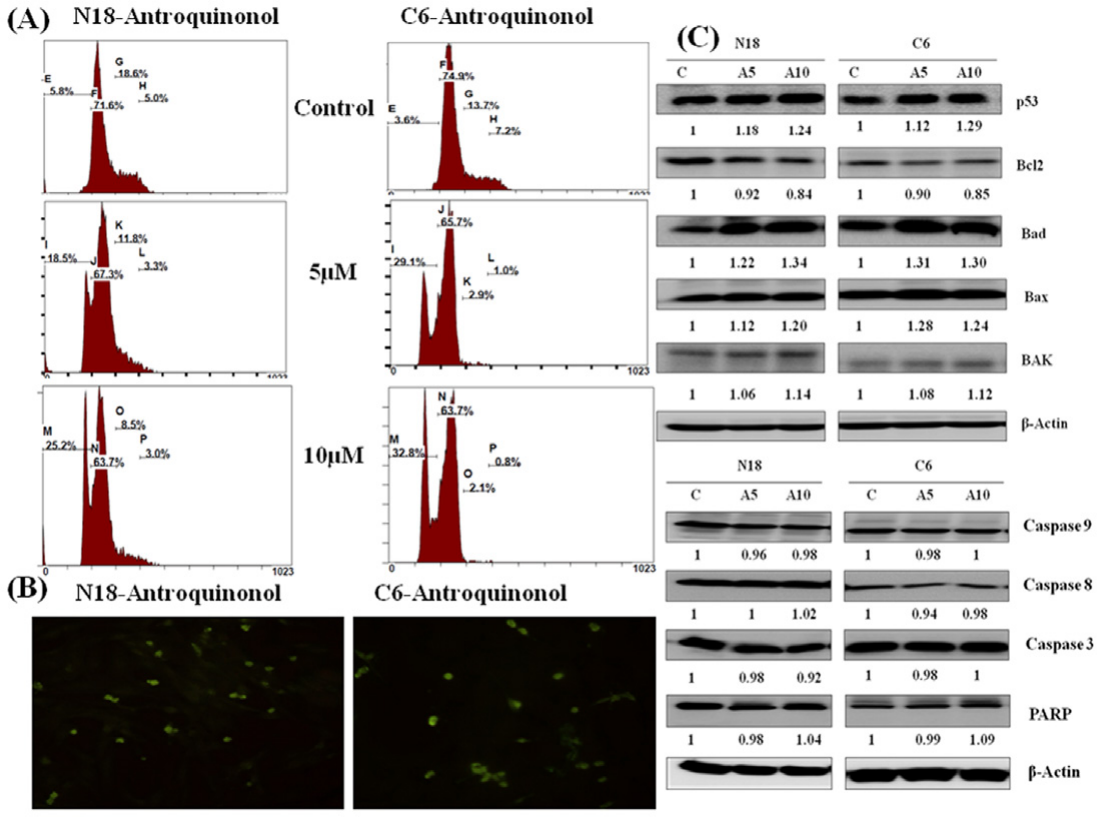
Effects of antroquinonol on pro-apoptotic & anti-apoptotic proteins. Cells were treated with the specified concentrations of antroquinonol for 24 h. (A) the cell-cycle distribution was analyzed by flow cytometry and propidium iodide (PI) staining. The figures show a representative staining profile for 10,000 cells per experiment. (B) Determination of apoptotic cells by TUNEL staining by green fluorescence. The morphological features were analyzed with an optical microscope at 100-fold magnification. (C) Cells were harvested and lysed for western blot analysis. Changes in the levels of p53, Bcl-2, Bad, Bax, Bak, and caspase 9, 8, 3, and PARP proteins after normalization to the levels of bet- actin are shown below each blot. Experiments were repeated three times with similar results. C—Control and A—Antroquinonol.

The cell-cycle distribution was investigated by flow cytometry analysis. Cells exposed to antroquinonol for 24 h showed a cell cycle arrest at the sub-G1 phase in a dose-dependent manner compared with the untreated cells (Table 1; Fig 2A). Antroquinonol-induced apoptosis in C6 and N18 cells was further determined with a TUNEL assay. Antroquinonol increased the accumulation of green fluorescent apoptotic cells after 24 h treatment (Fig 2B). This evidence reveals that antroquinonol caused the apoptosis of C6 and N18 cells.

**Table 1:** Changes of cell cycle progression by Antroquinonol in N18 and C6 cells.

Cell	Dosage (μM)	Sub G_1_ (%)	G_1_ (%)	S (%)	G_2_-M (%)
**N18**	Control	5.8± 1.32	71.6±1.20	18.6±1.23	5.0±1.36
	A-5	18.5±0.89**	67.3±1.75	11.8±2.55*	3.3±2.06
	A-10	25.2±2.06***	63.5±2.27*	8.5±0.95**	3.0±1.16
**C6**	Control	3.6±0.42	74.9±2.68	13.7±2.14	7.2±0.58
	A-5	29.1±1.20**	65.7±1.29	2.9±1.89**	1.0±0.71**
	A-10	32.8±1.83***	63.7±2.47*	2.1±1.54**	0.8±1.20**

Cells were treated with different concentrations of antroquinonol for 24 h. The distribution of cells at each phase of cell cycle was analyzed by flow cytometry. The data represent the mean ± SE from three independent experiments.

*p < 0.05,

**p < 0.01, and

***p < 0.001, compared with the untreated control.

### Alterations of pro-apoptotic and anti-apoptotic proteins

The effects of antroquinonol on the expression of apoptotic proteins in N18 and C6 cell lines were determined. The expression of Bad, Bax, Bak, and p53 were significantly increased while the expression of Bcl-2 was decreased after 24 h of antroquinonol treatment (Fig 2C). The effect of antroquinonol on the expression of caspase and PARP, two apoptosis associated proteins were further analyzed. Unexpectly, antroquinonol did not cause any change in the cleavage of caspase and PARP proteins in both cell lines. These results demonstrate that antroquinonol induced apoptosis through the down-regulation of Bcl-2.

### Effects of FAK/Src signaling pathway

**Fig 3:**
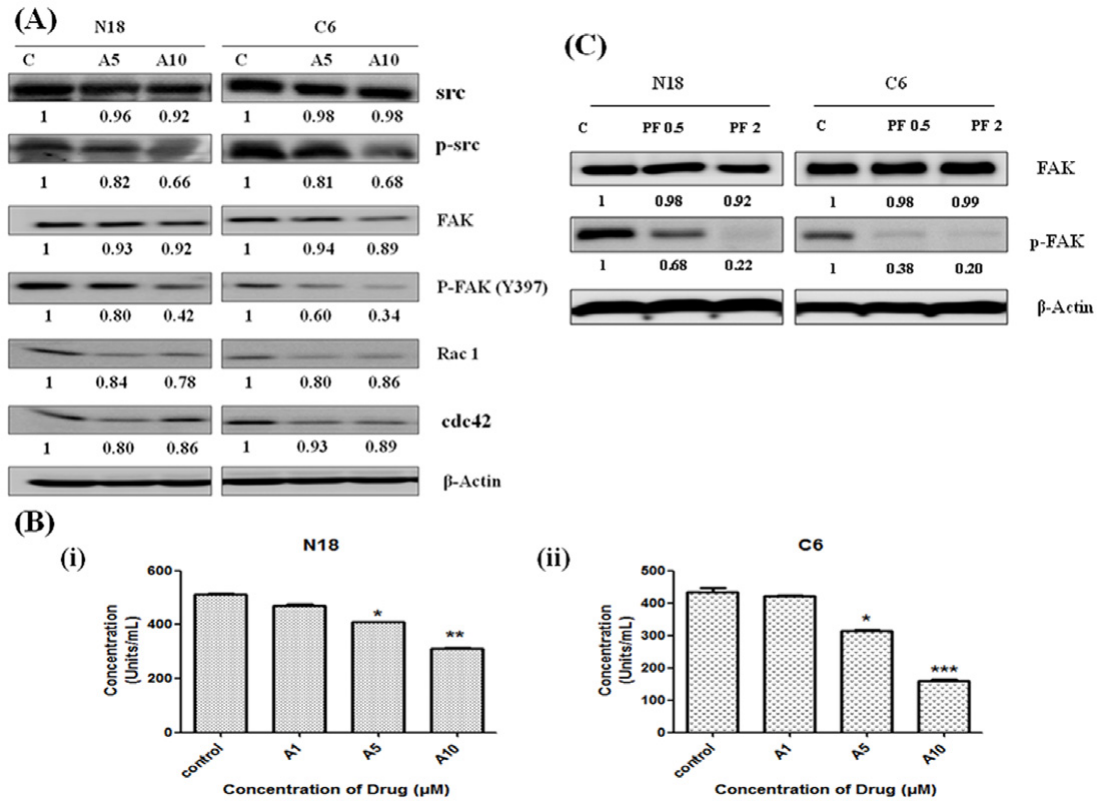
Effects of antroquinonol on FAK signaling pathway. Cells were treated with the indicated concentrations of antroquinonol (5 and 10 μM) for 24 h. Cells were harvested and lysed for western blot analysis. (A) Changes in the levels of FAK, pFAK, Src, pSrc, Cdc42, and Rac1 proteins after normalization to the levels of beta-actin are shown below each blot. (B) The *in vitro* assay was performed to detect the effect of antroquinonol on pFAK (Y397) (i) N18 and (ii) C6. (C) Cells were treated with PF 431396 (0.5 and 2 μM) for 24 h. Proteins were extracted and subjected to western blot analysis for FAK and pFAK. The results shown are the mean ±SEM of three independent experiments. *p < 0.05, **p < 0.01, and ***p < 0.001 when compared with the untreated controls. C—Control, A—antroquinonol and PF- PF 431396 (FAK inhibitor).

FAK, Src, Cdc42, and Rac1 are involved in the mediation of actin cytoskeleton remodeling, invasion, and cell migration. The expression of these proteins was measured by Western blot analysis. The protein levels of FAK, pFAK, Src, pSrc, Rac1, and Cdc42 were decreased after antroquinonol treatment for 24 h (Fig 3A). The results also showed that treatment with antroquinonol significantly decreased the phosphorylation of Y397 in a dose-dependent manner when compared with the control in both N18 and C6 cells (Fig 3Bi and 3Bii). These data illustrate that antroquinonol might regulate pFAK. The PF 431396 (FAK inhibitor) was applied as a reference control to verify that antroquinonol acted as a specific pFAK (Y397) inhibitor, and it effectively blocked the autophosphorylation of pFAK (Y397) (Fig 3C). These data illustrate that antroquinonol might regulate pFAK.

### Molecular docking and validation of FAK

**Fig 4:**
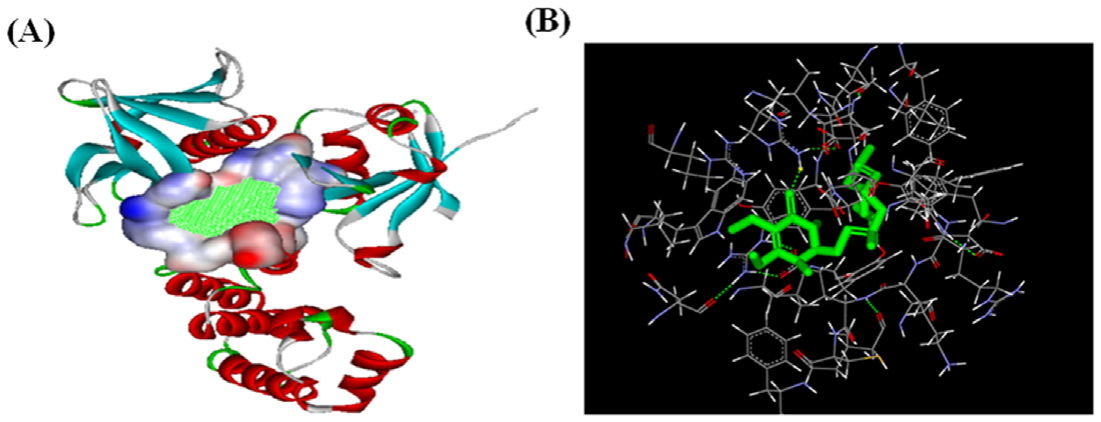
Targeting the Y397 site of FAK with a structure-based molecular docking approach. (A) The structure of FAK (FERM domain, PDB code 2AL6), and (B) antroquinonol.

To verify that antroquinonol acted as a pFAK (Y397) inhibitor, we computed the molecular docking to investigate the interaction of antroquinonol and FAK and furthermore examine the effect of antroquinonol on pFAK (Y397) with a pFAK (Y397) activity assay. The binding modes in the active sites were investigated with the Discovery Studio software (Fig 4A). From the interaction mode of the antroquinonol with the predicated active site, it has been noted that antroquinonol has a hydrogen bond donor interaction with Arg 86. Additionally, the oxygen of the carbonyl group of the cyclohexenone ring (antroquinonol) is responsible for the hydrogen bonding interaction with the components of the binding pocket (Fig 4B). These data confirm that antroquinonol indeed acted as a specific pFAK (Y397) inhibitor.

### Nano-morphological changes by AFM

**Fig 5:**
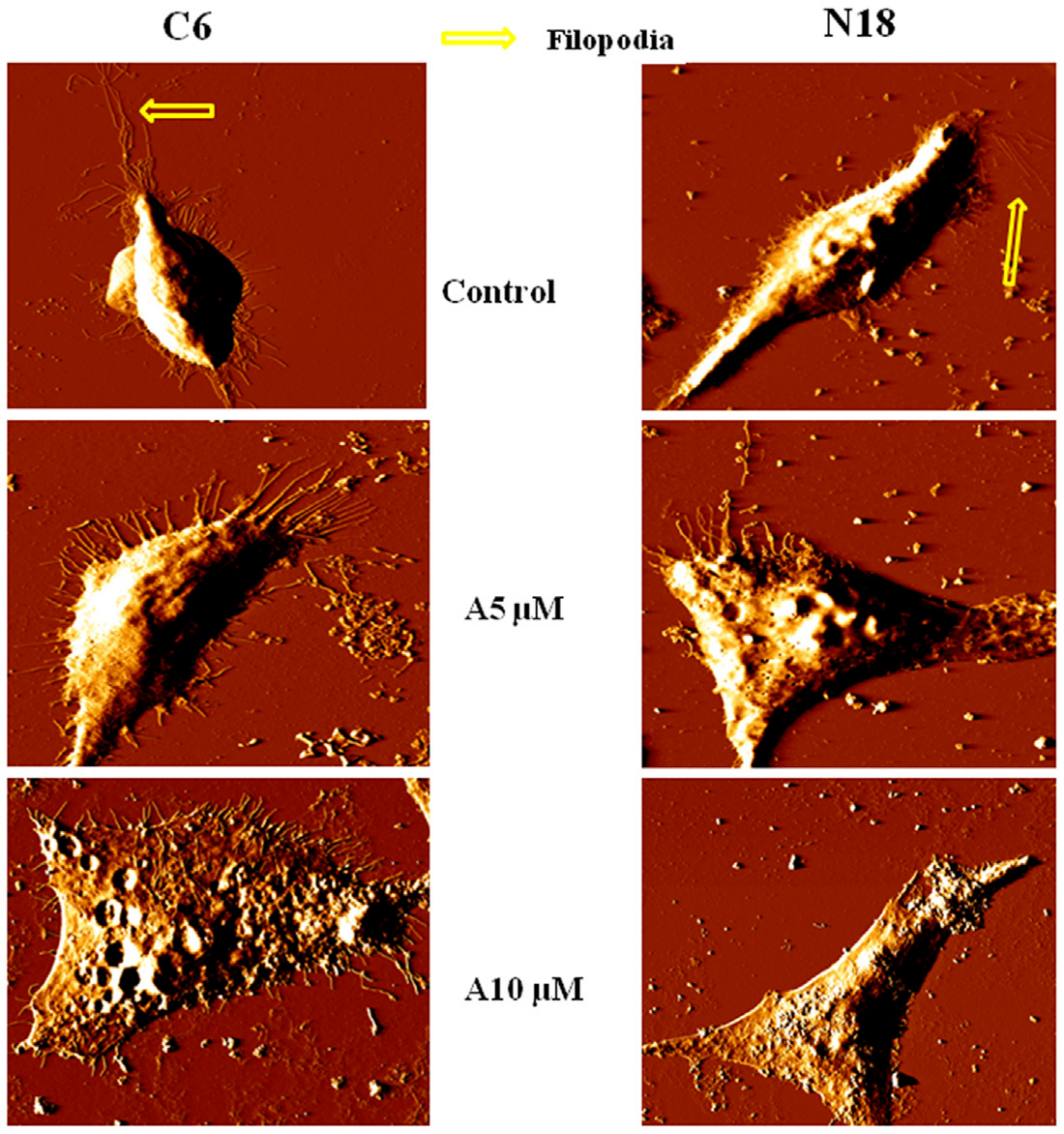
AFM of C6 and N18 cells after antroquinonol treatment. Cells were grown on coverslips and treated with various concentrations (5 and 10 μM) of antroquinonol for 24 h, and then fixed with 1% glutaraldehyde for 5 min. An AFM probe was scanned across the cell surfaces at the rate of 0.5 to 2 Hz with an area of approximately 100 μm x 100 μm. Representative images were taken by the AFM, and the images were collected using JPK Software. C—Control and A—antroquinonol.

Furthermore, to better understand how the compound may alter the ultra-structural cell morphology of cancer cells, atomic force microscopy (AFM) was used to measure the effect of antroquinonol on the morphology of C6 and N18 cells treated with 5 and 10 μM of antroquinonol. From the deflection image of the AFM (Fig 5), we observed that the compound increased the surface roughness of the cells and pore formations. Additionally, 10 μM of antroquinonol diminished the formation of filopodia when compared with the control cells. It seems that the anti-tumorigenic activity of antroquinonol abrogates the morphological structure of cancer cells.

### Effects of EMT markers

**Fig 6:**
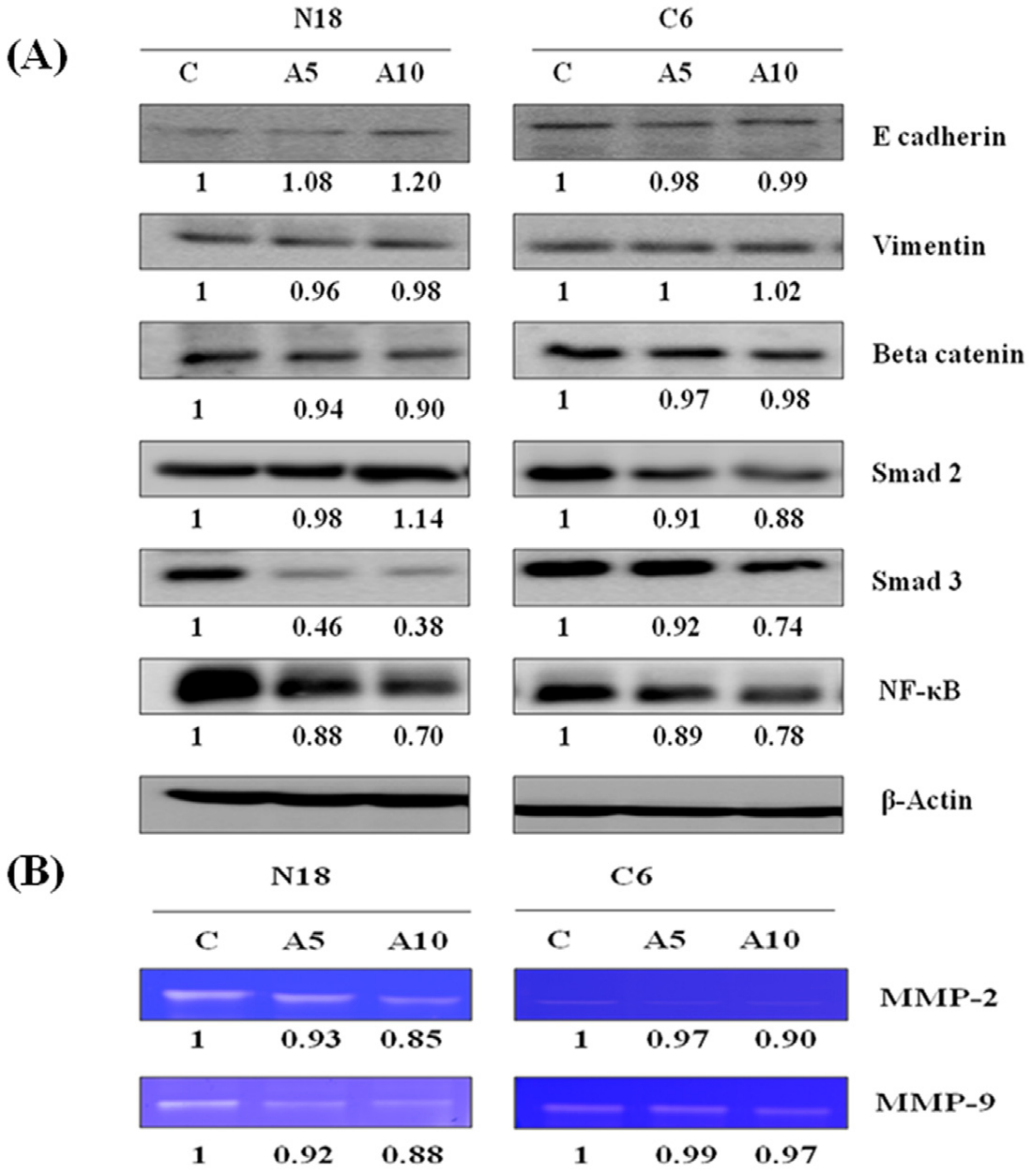
Effects of antroquinonol on EMT and MMP proteins in C6 and N18. Cells were treated with the indicated concentrations of antroquinonol (5 and 10 μM) for 24 h. (A) Cells were harvested and lysed for western blot analysis. Changes in the levels of E-cadherin, vimentin, NF-kB, beta-catenin, and Smad 2 and 3 proteins after being normalized to the levels of beta-actin are shown below each blot. (B) The supernatant of treated groups was collected for this assay. The MMP-2 and MMP-9 activities were measured using zymography and normalized with the control. These experiments were independently triplicated and similar results were obtained. C—Control and A—Antroquinonol.

Next, the effect of antroquinonol on epithelial-to-mesenchymal transition (EMT) markers was examined. After a 24 h treatment, antroquinonol increased the expression of E-cadherin in N18 and markedly decreased the protein levels of NF-kB, Smad2, and Smad3 in both N18 and C6 cells when compared with the untreated controls. Furthermore, antroquinonol treatment did not show any significant difference in protein levels of β-catenin and vimentin in either N18 or C6 cells (Fig 6A).

### Alterations of MMP expression

The effects of antroquinonol on the MMP family were examined. The cells were treated with various concentrations of antroquinonol for 24 h. The supernatant was collected and analyzed by gelatin zymography. After antroquinonol treatment, MMP2 and MMP9 activities were reduced in N18 cells, but no significant change in MMP9 activity was seen in the C6 cell line (Fig 6B).

### Antroquinonol inhibited tumor growth in nude mice

**Fig 7:**
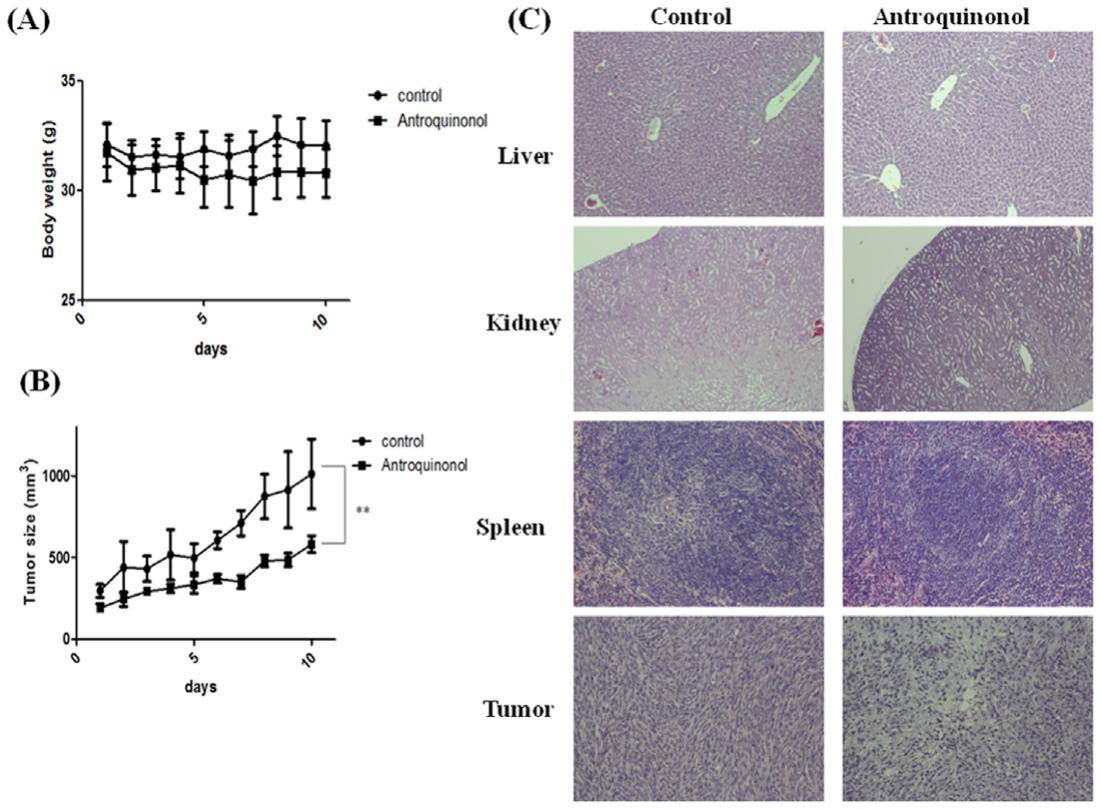
Anti-tumor efficacy of antroquinonol on nude mice bearing glioma xenograft (n = 6). (A) Tumor size, (B) body weight of the mice, and (C) H&E staining of paraffin-embedded tissue sections of the liver, kidney, spleen, and tumor (magnification 100X). The values are represented as the mean ± SD of three individual experiments. **p < 0.01 when compared with the untreated controls.

Subcutaneous C6 rat glioma cells were induced in nude mice as described in the methods section to test the *in vivo* efficacy of antroquinonol. Antroquinonol treatment significantly decreased the tumor volume when compared to the controls (Fig 7A) at day 10 of drug treatment. Furthermore, no difference in the body weight was found after antroquinonol treatment (Fig 7B). Major organs such as the liver, kidneys, and spleen, as well as tumors were stained with hematoxylin and eosin (H&E) and analyzed histologically. No tissue damage in the liver, kidneys, or spleen was detected after antroquinonol treatment. However, antroquinonol-treated tumor micro-sections revealed large areas of cell death when compared to the controls (Fig 7C). Biochemical analysis showed no significant differences in creatinine, amino transferase (ALT), and aspirate amino transferase (AST) levels after antroquinonol treatment (data not shown). These data indicate that antroquinonol could suppress tumor growth and that these doses of antroquinonol treatment were safe and non-toxic.

## Discussion

In this study, we investigated the effect of antroquinonol on cancer cells (mainly focused on C6 glioma cells), and subsequently tested antroquinonol on N18 neuroblastoma cells to confirm its anti-cancer properties and possible molecular mechanisms of action. The results demonstrated that antroquinonol was cytotoxic in both cell lines with IC50 values of 10 μM. Antroquinonol shows a significant inhibition of cell viability in both the N18 and C6 cell lines, which exhibited cell cycles in the sub-G1 phase suggesting an induction of apoptosis. Apoptosis is a major mechanism of cell death in response to various cancer therapies and is characterized by morphological events such as cell shrinking, DNA fragmentation, and fragmentation into membrane bound apoptotic bodies [29]. Antroquinonol treatment induced a significant accumulation of an apoptotic-portion sub-G1 peak as well as an increase of green TUNEL-positive apoptotic bodies in both C6 and N18 cells. Furthermore, AFM was used to measure the cells treated with antroquinonol to have a better understanding of how the compound altered the ultra-structural cell morphology of cancer cells. Cells were treated with various concentrations of antroquinonol and showed signs of apoptosis. Cells also displayed shrinking, disruption of the cytoskeleton, nucleus fragmentation, membrane blebbing, or slightly and peripheral chromatin condensation. This result is consistent with our previous study in which Kumar et al. observed structural and morphological changes in A549 cells after antroquinonol treatment [23]. Lamellipodia and filopodia are cytoskeletal actin structures that are involved in cell mobility. Filopodia formation is activated by Cdc42 while Rac1 promotes the formation of lamellipodia [30]. FAK/Src complex activates several pathways that lead to protruding activity via Rac and Cdc42 GTPases at sites of integrin ligation. Our data showed that antroquinonol treatment greatly suppressed the activity of FAK, pFAK, Src, and pSrc proteins. Molecular docking is a computation for molecular recognition and produces a fast prediction of the structures of the protein—ligand complex, particularly for structure-based drug designs [31]. Our direct predicted-binding model showed that antroquinonol docked in the Y397 site of FAK, which suggests a decrease in the phosphorylation level and some degree of specificity of antroquinonol towards this enzyme. Furthermore, Rac1 and Cdc42, which are the key regulators of cell adhesion and spreading, were substantially inhibited by antroquinonol in this study.

Bcl-2 family proteins have a demonstrated role in the process of apoptosis. Two different types of Bcl-2 family proteins have been identified: (1) pro-apoptotic proteins such as Bax, Bak, and Bcl-Xs, and (2) anti-apoptotic proteins such as Bcl-2, Bcl-XL, and Mcl-1. Previous studies have indicated that an increase in pro-apoptotic Bcl-2 family proteins and a decrease in anti-apoptotic Bcl-2 family proteins participate in apoptosis [32, 33]. In addition, the over-expression of anti-apoptotic Bcl-2 proteins can protect cells from stimulant-induced apoptosis [34]. p53 is a tumor suppressor protein and transcription factor that plays an important role in apoptosis and it can induce apoptosis by activating the pro- apoptotic protein Bax [35]. Antroquinonol treatment increased pro-apoptotic proteins and decreased Bcl-2, without showing any changes in caspase levels. Antroquinonol induced apoptotic cell death in both N18 and C6 cells through a caspase-independent mechanism and was associated with a reduction in the Bcl-2. This demonstrates that antroquinonol-induced apoptosis is mediated by a p53- and caspase-independent pathway. Our findings differ from a previous report [23] that suggest antroquinonol induces a caspase-dependent apoptosis. FAK has been shown to be important for survival signaling, angiogenesis, motility, and metastasis and has been shown to be overexpressed in a number of tumor cells [36]. Moreover, FAK has been proposed as a new potential therapeutic target for cancer [37, 38]. Recently, deletion of FAK promotes p53-mediated DNA damage in advanced squamous cancer cells [39] and the localization of apoptotic inducing factor (AIF) from mitochondria to nucleus leads to DNA fragmentation and regulates the caspase-independent apoptotic pathway [40]. We speculate that antroquinonol could induce apoptosis in both N18 neuroblastoma and C6 glioma cells by the nuclear translocation of AIF rather than through caspase activation; however, further investigation is necessary.

Losses of expression of epithelial adherens junction protein (E-cadherin) with a concomitant gain of mesenchymal marker expression (vimentin) are distinctive events in EMT and are common in metastatic carcinomas [41, 42]. FAK and Src play a critical role in tumors associated with EMTs promoting intracellular signaling pathways that lead to the induction of E-cadherin repressors and to the subsequent down-regulation of E-cadherin to allow tumor cell migration and invasion [43]. Src and FAK also induce cytoskeletal reorganization, causing the dissociation of E-cadherin from the membrane, loss of epithelial morphology, and increased cell motility [44]. Here, we demonstrate that antroquinonol treatment elicits E-cadherin induction and suppresses the expression of NF-kb, Smad2, and Smad3. Matrix metalloproteases (MMPs) play an important role in degrading ECM components. FAK siRNA dramatically decreased MMP9 and MMP2 at both the mRNA and protein levels in SMMC7721 and SK-hep1 cells [45]. FAK plays a pivotal role in the regulation of MMP2 and/or MMP9, which are considered to be critical for cancer metastasis and invasion [46]. The present results have shown that antroquinonol also decreases MMP2 and MMP9 protein levels, suggesting that antroquinonol possesses anti-invasive and anti-migratory properties.

*In vitro* studies have shown that antroquinonol is more potent in C6 and N18 cell lines. We also confirmed the anti-tumorigenic activity of antroquinonol *in vivo*. In mouse xenograft models with C6 glioma cells, antroquinonol significantly inhibited tumor growth and prolonged the doubling time of the tumor. Furthermore, antroquinonol treatment showed no significant body weight loss or tissue damage. These data indicate that antroquinonol has no toxic effects and possesses an anti-tumorigenic activity against glioma in both *in vitro* and *in vivo* assays.

In conclusion, this study demonstrated that antroquinonol has a potential effect on cell viability and induces apoptosis. Antroquinonol also suppresses FAK/Src complex formation, which subsequently inhibits Rac1 and Cdc42 activation. In addition, antroquinonol alters the expressions of EMT proteins and effectively reduces tumor volume in a xenograft mouse model. Thus, antroquinonol suppresses tumor proliferation through FAK inhibition, which suggests that antroquinonol could be a promising anti-tumor drug.
